# Bioenergetic Phenotyping of DEN-Induced Hepatocellular Carcinoma Reveals a Link Between Adenylate Kinase Isoform Expression and Reduced Complex I-Supported Respiration

**DOI:** 10.3389/fonc.2022.919880

**Published:** 2022-06-08

**Authors:** Kelsey L. McLaughlin, Margaret A.M. Nelson, Hannah S. Coalson, James T. Hagen, McLane M. Montgomery, Ashley R. Wooten, Tonya N. Zeczycki, Nasreen A. Vohra, Kelsey H. Fisher-Wellman

**Affiliations:** ^1^ Brody School of Medicine, Department of Physiology, East Carolina University, Greenville, NC, United States; ^2^ East Carolina Diabetes and Obesity Institute, East Carolina University, Greenville, NC, United States; ^3^ Brody School of Medicine, Department of Biochemistry and Molecular Biology, East Carolina University, Greenville, NC, United States; ^4^ Brody School of Medicine, Department of Surgery, East Carolina University, Greenville, NC, United States; ^5^ UNC Lineberger Comprehensive Cancer Center, University of North Carolina at Chapel Hill School of Medicine, Chapel Hill, NC, United States

**Keywords:** metabolism, hepatocellular carcinoma, mitochondria, cancer, bioenergetics

## Abstract

Hepatocellular carcinoma (HCC) is the most common form of liver cancer worldwide. Increasing evidence suggests that mitochondria play a central role in malignant metabolic reprogramming in HCC, which may promote disease progression. To comprehensively evaluate the mitochondrial phenotype present in HCC, we applied a recently developed diagnostic workflow that combines high-resolution respirometry, fluorometry, and mitochondrial-targeted nLC-MS/MS proteomics to cell culture (AML12 and Hepa 1-6 cells) and diethylnitrosamine (DEN)-induced mouse models of HCC. Across both model systems, CI-linked respiration was significantly decreased in HCC compared to nontumor, though this did not alter ATP production rates. Interestingly, CI-linked respiration was found to be restored in DEN-induced tumor mitochondria through acute *in vitro* treatment with P1, P5-di(adenosine-5′) pentaphosphate (Ap5A), a broad inhibitor of adenylate kinases. Mass spectrometry-based proteomics revealed that DEN-induced tumor mitochondria had increased expression of adenylate kinase isoform 4 (AK4), which may account for this response to Ap5A. Tumor mitochondria also displayed a reduced ability to retain calcium and generate membrane potential across a physiological span of ATP demand states compared to DEN-treated nontumor or saline-treated liver mitochondria. We validated these findings in flash-frozen human primary HCC samples, which similarly displayed a decrease in mitochondrial respiratory capacity that disproportionately affected CI. Our findings support the utility of mitochondrial phenotyping in identifying novel regulatory mechanisms governing cancer bioenergetics.

## Introduction

Hepatocellular carcinoma (HCC) is the most common form of primary liver cancer worldwide and is among the top 5 causes of cancer-related death in men of all ages in the United States (1, 2). Although early detection and resection of small neoplasms may improve 5-year survival to above 50%, the most recent U.S. estimates put the average 5-year survival at around 20%, making liver cancer the second deadliest cancer behind pancreatic cancer (1, 3). The burden of this disease is likely to continue to grow as HCC commonly develops in the context of pre-existing chronic liver disease (i.e., hepatitis, cirrhosis) and metabolic diseases (i.e., fatty liver disease, diabetes), the latter of which remains a growing health issue for Americans (2).

Emerging evidence from human, rodent, and cell models of HCC suggests that mitochondrial reprogramming plays an important role in HCC metabolism and disease progression (4, 5). HCC tumors commonly present with marked upregulation of glycolytic enzymes and metabolites (6–8), decreased mitochondrial DNA (mtDNA) copy number (9, 10), and increased mtDNA mutational burden (11–14). Together, these findings are suggestive of a metabolic phenotype in which mitochondrial respiration is impaired to promote an increase in glycolytic energy metabolism despite sufficient oxygen availability, commonly described in cancer metabolism as the Warburg effect (15). Further, a recent longitudinal, multi-omic study in a rat model of HCC induced by the carcinogen diethylnitrosamine (DEN) has demonstrated that changes to mitochondrial metabolism precede tumor formation, suggesting that mitochondrial reprogramming is integral to the tumorigenic process (5).

Despite this growing interest in mitochondria, most studies of HCC metabolism to date have relied upon broad genomic, proteomic, or metabolomic assessments (5, 16–18). As a result, there is a dearth of literature describing the functional consequences of these metabolic changes in the mitochondria, and the few studies that have assessed aspects of mitochondrial function have been limited in scope. For instance, evaluations of mitochondrial respiration in DEN-induced rodent models of HCC have almost exclusively been limited to comparisons of mitochondrial respiratory control ratios between healthy and malignant livers (19, 20). In some cases, mitochondrial function was extrapolated solely from measurements of the individual activities of TCA cycle enzymes or mitochondrial respiratory complexes (21, 22). Additionally, DEN-induced tumors were not isolated from the surrounding tissue before assessment, preventing any comparisons between tumor and nontumor mitochondria within the same animal (19–22). These experimental limitations make it difficult to interpret the functional implications of previously reported differences in HCC and to define which intrinsic properties of mitochondria are altered to support tumor metabolism.

We sought to address this gap in the field by applying a comprehensive diagnostic workflow developed by our lab group that combines high-resolution respirometry, fluorometry, and mitochondrial-targeted nLC-MS/MS proteomics (23, 24). This workflow was recently applied to several models of acute myeloid leukemia and revealed that leukemic mitochondria have an intrinsic limitation in producing ATP through oxidative phosphorylation (25). In the present study, we first applied an abbreviated version of the workflow to mitochondria isolated from mouse HCC-derived cells (Hepa 1-6) and a nontumorigenic mouse hepatocyte cell line (AML12). We then expanded this work using a DEN-induced model of HCC in C3H mice in which isolated mitochondria from tumor and nontumor liver tissues were subjected to the full battery of assays including assessments of mitochondrial respiration, membrane potential (ΔΨ), NAD^+^/NADH redox poise, ATP production rates, calcium retention, and mass spectrometry-based proteomics. Finally, human primary HCC samples were obtained for comparisons of mitochondrial respiratory capacity in freeze-fractured mitochondria. Overall, our data highlight that most measures of maximal mitochondrial capacity were comparable between HCC and hepatocytes, but our diagnostic workflow was able to highlight more nuanced differences that may be physiologically relevant to HCC metabolism.

## Results

### Cells Derived From Hepatocellular Carcinoma Display a Profound Reduction in Complex I-Supported Respiration

**Figure 1:**
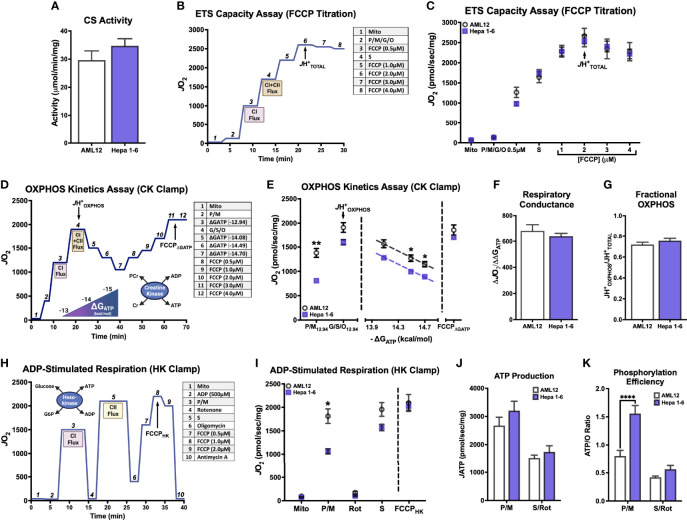
Hepatocellular carcinoma-derived (Hepa 1-6) cells display profound impairment in complex I-supported respiration compared to nontumorigenic immortalized hepatocytes (AML12). All experiments were performed using isolated mitochondria and normalized to mitochondrial protein (mg). **(A)** Citrate synthase (CS) activity. **(B)** Schematic depicting oxygen consumption (*J*O_2_) during the ETS Capacity Assay (FCCP titration) where point 3 represents FCCP-stimulated flux in the presence of complex I substrates pyruvate/malate (P/M), point 4 represents FCCP-stimulated flux in the presence of complex I and II substrates P, M, glutamate, succinate, octanoyl-L-carnitine (P/M/G/S/O), and point 6-7 represents the maximal proton conductance of the electron transport system (*J*H^+^_TOTAL_). **(C)** ETS capacity protocol measured in AML12 and Hepa 1-6. **(D)** Schematic depicting *J*O_2_ during the OXPHOS Capacity Assay (ΔG_ATP_ titration) where point 3 represents ΔG_ATP_-stimulated flux with P/M, point 4 represents ΔG_ATP_-stimulated flux with P/M/G/S/O or the maximal proton conductance by the OXPHOS system (*J*H^+^_OXPHOS_), and point 11-12 represents the maximum proton conductance in the presence of ΔG_ATP_ (FCCP_ΔGATP_). **(E)** OXPHOS capacity protocol measured in AML12 and Hepa 1-6. **(F)** Comparison of respiratory conductance from dotted lines in **(E)**. **(G)** Comparison of fractional OXPHOS calculated as the ratio of *J*H^+^_OXPHOS_ to *J*H^+^_TOTAL_. **(H)** Schematic depicting *J*O_2_ during the ADP-stimulated Respiration where point 3 represents ADP-stimulated flux with P/M, point 5 represents ADP-stimulated flux with S/rotenone (Rot), and point 8 represents maximal proton conductance in the presence of ADP and oligomycin (FCCP_HK_). **(I)** ADP-stimulated protocol with AML12 and Hepa 1-6. **(J)** ATP production rate (*J*ATP) fueled by P/M and S/Rot. **(K)** Phosphorylation efficiency, quantified as the ATP/O ratio, fueled by P/M and S/Rot. Data are presented as mean ± SEM and analyzed by unpaired t-test **(A, F, G)** and two-way ANOVA (C, E, I–K). *p < 0.05, **p < 0.01, ****p < 0.0001.

We first sought to define the bioenergetic impact of hepatocellular carcinoma (HCC) malignancy using commercially available cell lines derived from mouse HCC (Hepa 1-6) and healthy, immortalized hepatocytes (AML12). All experiments were performed in isolated mitochondria, and the purity of these mitochondrial isolations was found to be comparable using the citrate synthase (CS) activity assay, a validated surrogate for hepatic mitochondrial content (26) (**Figure 1A**). Three unique respiratory stimuli—chemical uncoupling (**Figure 1B**), manipulation of ATP free energy (ΔG_ATP_; **Figure 1D**), and supramaximal ADP (**Figure 1H**)—were then used to evaluate specific aspects of mitochondrial function.

First, the maximal respiratory capacity of the electron transport system (ETS) was determined through titration of the uncoupler carbonyl cyanide-4-phenylhydrazone (FCCP), as depicted in **Figure 1B**. Saturating amounts of carbon substrates were provided sequentially such that pyruvate (P), malate (M), glutamate (G), and octanoyl-L-carnitine (O)—all of which primarily introduce electrons into the ETS at complex I (CI)—were added together, and the complex II (CII)-specific substrate succinate (S) was added following the first FCCP titration point (**Figure 1B**). This allowed for comparison of CI-supported oxygen consumption (*J*O_2_) in addition to that supported by the whole ETS within the same assay. Both CI-linked *J*O_2_ and the maximal FCCP-stimulated rate (*J*H^+^_TOTAL_) were comparable between AML12 and Hepa 1-6 mitochondria (**Figure 1C**), suggesting similar ETS capacities.

We then compared the ability of AML12 and Hepa 1-6 mitochondria to kinetically respond to a more physiological stimulus for respiration: ΔG_ATP_, or the demand for ATP resynthesis through oxidative phosphorylation (OXPHOS). Using the creatine kinase (CK) energetic clamp (**Figure 1D**), we titrated ΔG_ATP_ across a physiological span from maximal ATP demand (ΔG_ATP_ = -12.94kcal/mol) to minimal demand (ΔG_ATP_ = -14.7kcal/mol). As in the ETS capacity assay, carbon substrates were added sequentially (**Figure 1D**), allowing for separate measurements of CI-supported (P/M) and whole ETS-supported (P/M/G/S/O) respiration. The *J*H^+^_OXPHOS_ corresponded to the maximal *J*O_2_ stimulated by ΔG_ATP_ in the presence of all substrates. Interestingly, the maximal ΔG_ATP_-stimulated rate fueled by CI substrates P/M alone was decreased in Hepa 1-6 mitochondria compared to AML12 (‘P/M_12.94_’, **Figure 1E**), but this difference was no longer significant when additional substrates were added (‘G/S/O_12.94_’, **Figure 1E**).

When looking at the respiratory response to changes in ΔG_ATP_, absolute *J*O_2_ was lower in Hepa 1-6 mitochondria toward the end of the titration. However, the respiratory conductance, quantified as the slope of the linear portion of the *J*O_2_/ΔG_ATP_ relationship (dotted lines, **Figure 1E**), was not significantly different between AML12 and Hepa 1-6 mitochondria (**Figure 1F**). This suggests that both groups are equally able to kinetically adjust oxygen consumption to ATP demand. Following the titration of ΔG_ATP_, we again titrated the uncoupler FCCP to assess whether there were any inhibitory effects of the presence of ΔG_ATP_ on ETS flux, which we had previously observed in leukemic mitochondria (25)(**Figure 1D**). For simplicity, only the maximal FCCP-stimulated rate under these conditions was graphed as the FCCP_ΔGATP_ (**Figures 1D, E**). Both AML12 and Hepa 1-6 mitochondria were able to attain a *J*O_2_ equivalent to their *J*H^+^_OXPHOS_ when FCCP was added at the end of the CK clamp assay, suggesting a lack of inhibition by ΔG_ATP_ for both cell types.

As both the ETS capacity and OXPHOS kinetics assays ultimately use the same combination of carbon substrates (P/M/G/S/O), we can compare the maximal *J*O_2_ that was stimulated by ATP demand (*J*H^+^_OXPHOS_) to the total respiratory capacity of the ETS elicited by FCCP (*J*H^+^_TOTAL_) as an estimate of how much of the total capacity is able to contribute to OXPHOS. We have previously termed this ratio ‘Fractional OXPHOS’ (25)(**Figure 1G**). Fractional OXPHOS was found to be equivalent between AML12 and Hepa 1-6 mitochondria, with both able to draw on approximately 74% of their *J*H_TOTAL_ in response to elevated ATP demand. This may reflect a small amount of inefficiency in both cell types that prevents them from fully activating all mitochondrial complexes when stimulated by ΔG_ATP_.

To further explore the CI limitation that was revealed in the OXPHOS kinetics assay and its potential impact upon mitochondrial ATP phosphorylation, we then incorporated an assay in which ADP-stimulated respiration and ATP production rate (*J*ATP) could be determined in parallel under identical assay conditions (**Figure 1H**). Mitochondria were incubated with a supraphysiological concentration of ADP (500μM), which was maintained using the hexokinase (HK) enzymatic clamp. Both CI-linked flux (P/M) and CII-linked flux (S) were assessed independently within the assay using the addition of CI inhibitor rotenone (Rot) after P/M. ADP-linked respiration was then inhibited by the addition of oligomycin, and FCCP was titrated to elicit the maximal S/Rot-supported *J*O_2_, here defined as FCCP_HK_ (**Figure 1H**). Once again, it was found that P/M-supported respiration was lower in Hepa 1-6 mitochondria than AML12 (‘P/M’, **Figure 1I**), but respiration was no longer different when fueled by S/Rot and stimulated by either ADP or FCCP (‘S’, ‘FCCP_HK_’, **Figure 1I**). Surprisingly, *J*ATP did not differ significantly between Hepa 1-6 and AML12 mitochondria under either substrate condition (**Figure 1J**), suggesting that although respiration was lower in Hepa 1-6 when fueled by P/M, the ability to phosphorylate ATP was retained. As a result, the efficiency of ATP production, defined as the amount of ATP produced per molecule of oxygen consumed, or ATP/O, was significantly higher for Hepa 1-6 mitochondria fueled by P/M (**Figure 1K**).

### Induction of Hepatocellular Carcinoma in Mice Using Diethylnitrosamine Results in Clearly Defined Tumors for Mitochondrial Isolation

**Figure 2:**
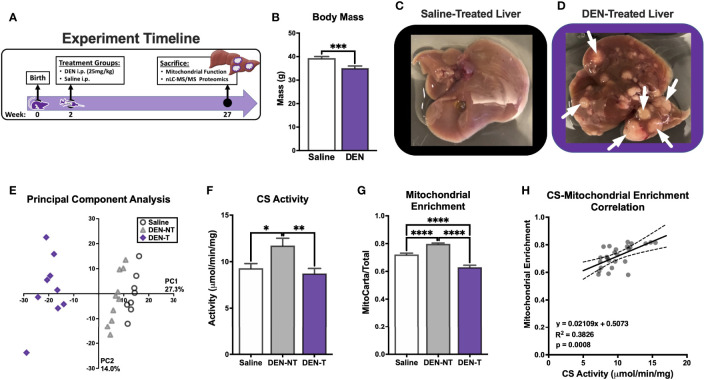
Induction of hepatocellular carcinoma in C3H mice using diethylnitrosamine (DEN) and the purity of the resultant isolated mitochondrial preparations. **(A)** Experiment timeline of DEN treatment. **(B)** Body weight (g) of saline and DEN-treated mice at time of sacrifice. **(C, D)** Images of saline **(C)** and DEN-treated livers **(D)**; white arrows denote tumors. **(E)** Principal component analysis (PCA) of isolated mitochondrial preparations from saline treated liver (Saline), DEN-treated nontumor (DEN-NT), and DEN-induced tumor (DEN-T). **(F)** Citrate synthase (CS) activity of isolated mitochondria. **(G)** Comparison of mitochondrial enrichment calculated of the ratio of mitochondrial (MitoCarta) to total protein abundance. **(H)** Linear correlation between CS activity and mitochondrial enrichment. Data are presented as mean ± SEM and analyzed by unpaired t-test **(B)** and one-way ANOVA **(F, G)**. *p < 0.05, **p < 0.01, ***p < 0.001, ****p < 0.0001.

To expand on this interesting finding of a potential limitation to CI-supported respiration in a more physiologically relevant model of HCC, we next employed a mouse model of HCC development that would allow mitochondrial isolation from fresh tumors. As depicted in **Figure 2A**, male C3H mice were given a single IP injection of diethylnitrosamine (DEN; 25mg/kg) or vehicle control (Saline) at postnatal day 14 (P14). Mice were sacrificed after tumor development at about 27 weeks of age. At the time of sacrifice, there was a small but significant decrease in body mass in DEN-treated mice compared to Saline (**Figure 2B**). Saline-treated livers had almost no visible abnormalities (**Figure 2C**), whereas DEN-treated livers displayed numerous, clearly demarcated tumors surrounded by nontumor liver tissue (white arrows point to tumors; **Figure 2D**).

For optimal experimental contrast, mitochondria were isolated from 3 distinct tissue types: saline-treated liver (referred to throughout as Saline), DEN-treated nontumor tissue (DEN-NT), and DEN-induced tumor tissue (DEN-T). The comparison of mitochondria from both tumor and nontumor tissues of DEN-treated mice allowed for the separation of intrinsic alterations that support tumor metabolism from the effects of drug exposure. A subset of mitochondrial preparations from these tissues were subjected to mitochondrial-targeted, label-free, quantitative nLC-MS/MS proteomics. Overall, 2,512 proteins were identified and quantified, and 810 of these were identified to be mitochondrial using the mouse MitoCarta 3.0 database (27). Supporting the effective separation of DEN-T from DEN-NT tissue during mitochondrial isolation, principal component analysis of the 3 groups revealed that DEN-T samples clustered together away from both DEN-NT and Saline samples, with DEN-NT samples nearly clustering with Saline (**Figure 2E**). Differences in protein expression between the mitochondrial proteomes of Saline, DEN-NT, and DEN-T preparations were also quantified (**Supplementary Figures 1A–C**).

As purity can affect the interpretation of mitochondrial outcome measures normalized to total or crude mitochondrial protein amount, we compared the purity of our mitochondrial isolations using two separate methods. First, mitochondrial purity was estimated using the CS activity assay. DEN-NT mitochondria had significantly greater CS activity than both DEN-T and Saline mitochondria (**Figure 2F**), though there was no difference between Saline and DEN-T. Higher CS activity would suggest a higher mitochondrial purity for DEN-NT isolations. To confirm this finding, we also empirically derived the mitochondrial enrichment factor (MEF) for each tissue by quantifying the summed abundances of all proteins identified to be mitochondrial using the MitoCarta 3.0 database relative to the total protein abundance, as previously described (26). Consistent with the CS activity assay, the MEF for DEN-NT mitochondria was significantly greater than both DEN-T and Saline (**Figure 2G**). DEN-T mitochondria also had a significantly lower MEF than Saline mitochondria (**Figure 2G**). In support of our previous findings in liver mitochondria (26), MEF was significantly correlated with CS activity (**Figure 2H**). Given the significant differences in the purity of mitochondrial isolations across tissues identified through both CS activity and MEF, all functional outcome measures were normalized to MEF unless otherwise specified.

### Broad Characterization of DEN-Induced Tumor Mitochondria Reveals Reduced Mitochondrial Membrane Potential as well as Increased Sensitivity to FCCP and Calcium

**Figure 3:**
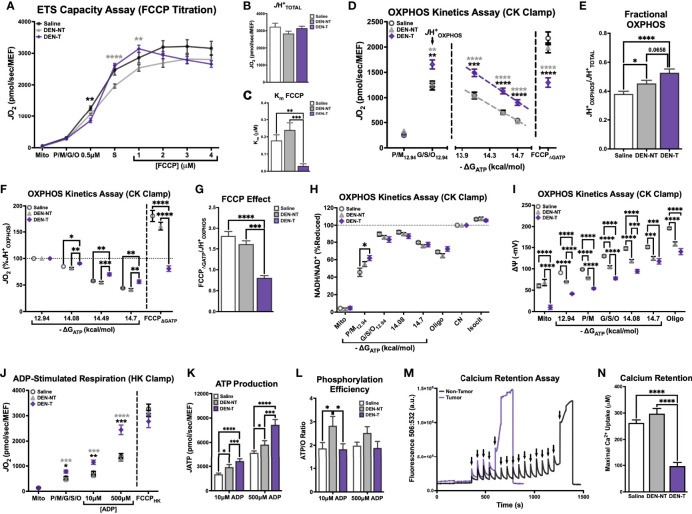
Broad characterization of DEN-induced tumor mitochondria across diverse biochemical stimuli. All experiments were performed using isolated mitochondria and normalized to mitochondrial enrichment factor (MEF). **(A)** ETS capacity protocol measured in Saline, DEN-NT, and DEN-T, with significant differences from DEN-T denoted by black asterisks for Saline, gray for DEN-NT. **(B)** Comparison of *J*H^+^_TOTAL_ from **(A)**. **(C)** Comparison of K_m_ of FCCP from **(A)**. **(D)** OXPHOS capacity protocol measured in Saline, DEN-NT, and DEN-T, significant differences from DEN-T denoted by black asterisks for Saline, gray for DEN-NT. **(E)** Comparison of fractional OXPHOS from **(D)**. **(F)** OXPHOS capacity protocol from **(D)**, graphed as % *J*H^+^_OXPHOS_. **(G)** Comparison of FCCP effect, quantified as the ratio of FCCP_ΔGATP_ to *J*H_OXPHOS_. **(H)** Relationship between ΔG_ATP_ and NAD^+^/NADH redox poise as % cyanide-induced reduction (CN). **(I)** Mitochondrial membrane potential across a ΔG_ATP_ span. **(J)** ADP-stimulated protocol measured in Saline, DEN-NT, and DEN-T fueled by P/M/G/S/O, significant differences from DEN-T denoted by black asterisks for Saline, gray for DEN-NT. **(K)** Comparison of *J*ATP when stimulated with 10μM and 500μM ADP. **(L)** ATP/O ratio when stimulated with 10μM and 500μM ADP. **(M)** Representative fluorometric trace of calcium retention assay (Ex/Em: 506/532); black arrows represent 30μM additions of calcium; sharp increase in fluorescence was considered MPTP opening. **(N)** Comparison of maximal calcium uptake (μM) prior to MPTP opening. Data are presented as mean ± SEM and analyzed by two-way ANOVA **(A, D, F, H–L)** and one-way ANOVA **(B, C, E, G, N)**. *p < 0.05, **p < 0.01, ***p < 0.001, ****p < 0.0001.

Before evaluating any complex-specific deficiencies, we first sought to characterize several aspects of general mitochondrial functionality, including respiration, NAD^+^/NADH redox poise, membrane potential (ΔΨ), ATP production (*J*ATP and ATP/O), and calcium retention. Total ETS capacity (*J*H^+^_TOTAL_) when fueled by saturating carbon substrates (P/M/G/S/O) was determined using an FCCP titration as described above (**Figure 1B**). Although the maximal FCCP-stimulated *J*O_2_ was not different between groups (**Figure 3B**), the shape of the FCCP titration curve was drastically different between mitochondria isolated from DEN-induced tumors and the two non-tumor tissues (**Figure 3A**). DEN-T mitochondria appeared more sensitive to smaller amounts of FCCP, as evident in their significantly lower K_m_ for FCCP compared to the other two tissues (**Figure 3C**).

OXPHOS kinetics were quantified using the CK clamp as described above (**Figure 1D**). The maximal *J*O_2_ stimulated by ΔG_ATP_ (*J*H^+^_OXPHOS_) was significantly higher for DEN-T mitochondria than both nontumor tissues, and *J*O_2_ remained significantly elevated in DEN-T mitochondria across the remainder of the ΔG_ATP_ titration (**Figure 3D**). Interestingly, fractional OXPHOS was significantly greater in mitochondria isolated from both DEN-treated tissues compared to Saline mitochondria (**Figure 3E**), and there was a trend for higher fractional OXPHOS in DEN-T mitochondria over DEN-NT (p=0.0658; **Figure 3E**). Taken alone, these data would imply that DEN-T mitochondria may be more robust than nontumor mitochondria as they appear to be able to contribute a higher fraction of their total respiratory capacity to OXPHOS. However, other outcomes from the OXPHOS kinetics assay suggest a more nuanced interpretation is necessary. For instance, the respiratory conductance calculated from the linear portion of the OXPHOS kinetics assay, which would commonly be considered a measure of sensitivity to dynamic changes in ΔG_ATP_, was equivalent between all 3 mitochondrial preparations (**Supplementary Figure 2A**). However, when the data was graphed as a percentage of the *J*H_OXPHOS_ (**Figure 3F**), DEN-T mitochondria did not proportionally reduce their respiration to the same degree as the nontumor mitochondria in response to changes to ΔG_ATP_.

Additionally, DEN-T mitochondria displayed a significantly reduced ability to respond to FCCP following ΔG_ATP_ titration (FCCP_ΔGATP_) compared to both nontumor tissues (**Figure 3F**), which may imply a degree of ETS inhibition in the presence of ΔG_ATP_. We have previously reported this phenomenon, termed the ‘FCCP effect’, in leukemia (25). As in leukemia, DEN-T mitochondria could not recover their *J*H^+^_OXPHOS_ respiration rate when stimulated by FCCP in the presence of ΔG_ATP_ whereas the FCCP_ΔGATP_ of nontumor mitochondria surpassed their *J*H^+^_OXPHOS_ (**Figure 3G**). Our previous work in leukemia had found that *in vitro* treatment of mitochondria with the heat shock protein 90 (HSP90) inhibitor 17AAG or the adenine nucleotide translocase (ANT) inhibitor carboxyatractyloside (CAT) would rescue the FCCP effect in leukemic cells (25). However, neither 17AAG nor CAT had any effect on the FCCP_ΔGATP_ of DEN-T (**Supplementary Figure 2B**). Interestingly, 17AAG did increase Fractional OXPHOS through an increase in *J*H_OXPHOS_ in both DEN-NT and Saline mitochondria, with no effect on DEN-T (**Supplementary Figures 2C–F**). The magnitude of this effect was similar in Saline and DEN-NT mitochondria despite only DEN-NT exhibiting a significant increase in the abundance of TRAP1, the purported mitochondrial target of 17AAG (**Supplementary Figure 2G**).

Parallel fluorometric experiments were conducted to compare additional driving forces underlying OXPHOS kinetics, NAD^+^/NADH redox poise (**Figure 3H**) and ΔΨ (**Figure 3I**), across the same ΔG_ATP_ titration. NAD^+^/NADH redox poise, expressed as a percentage of the maximal reduction induced by complex IV inhibitor cyanide (CN), was nearly identical between the 3 tissues when all 5 carbon substrates were present (‘G/S/O_12.94_’ to the end of the assay, **Figure 3H**). Interestingly, redox poise was significantly more reduced in DEN-T mitochondria compared to Saline when P/M was the sole carbon substrate (‘P/M_12.94_’, **Figure 3H**). Relative hyper-reduction may suggest a decreased ability of DEN-T mitochondria to transfer electrons from NADH into the ETS at CI. However, this observation is difficult to interpret because there was no difference in redox poise between the two DEN-treated tissues, and the effect was only present at this single point. In contrast, there were large differences in tetramethyl rhodamine methyl ester (TMRM)-derived ΔΨ between DEN-T and the two nontumor tissues across the ΔG_ATP_ span (**Figure 3I**). Saline mitochondria were able to drive the highest ΔΨ at each titration point compared to the two DEN-treated tissues, and DEN-T mitochondria were only able to match the DEN-NT ΔΨ at the final ΔG_ATP_ titration point (**Figure 3I**). The combination of high respiration and low membrane potential that was observed in DEN-T mitochondria across the ΔG_ATP_ span may indicate some degree of uncoupling of respiration from OXPHOS. Importantly, the mV values reported in **Figure 3I** were derived using tissue-specific TMRM standard curves, and these standard curves were significantly different from one another (**Supplementary Figure 2H**), a caveat that makes interpretation of this ΔΨ data difficult. However, it is possible that these tissue-specific differences were real, as there was considerable variability in the raw TMRM traces across tissues (**Supplementary Figure 2I**). Additionally, our group has previously found similar ΔΨ values across the same ΔG_ATP_ span in human HCC-derived HepG2 cells, supporting the possibility that this observation is not a result of an instrumental artifact (28).

To empirically test the coupling between respiration and ATP phosphorylation across tissues, a variation of the HK clamp-based assay described in **Figure 1H** was used. Mitochondria were energized with saturating carbon substrates (P/M/G/S/O), followed by a two-point ADP titration to the sub-K_m_ concentration of 10μM and the supraphysiological concentration of 500μM. The FCCP_HK_ rate was obtained by adding oligomycin to inhibit ADP-stimulated respiratory flux and titrating FCCP. DEN-T mitochondria exhibited significantly higher *J*O_2_ than both nontumor tissues following the addition of substrate alone (‘P/M/G/S/O’, **Figure 3J**), as well as both ADP titration points (**Figure 3J**). Interestingly, all 3 tissues displayed similar FCCP-stimulated respiration, suggesting that ADP is not as powerful as a stimulus as FCCP for Saline and DEN-NT mitochondria under this assay condition (**Figure 3J**). In a parallel fluorometric assay, *J*ATP was determined to be higher in DEN-T mitochondria than nontumor tissues at both ADP concentrations, and higher in DEN-NT mitochondria than Saline (**Figure 3K**). When phosphorylation efficiency was quantified from these two assays, all tissues had a comparable ATP/O ratio at 500μM ADP, but the ATP/O for DEN-NT was significantly greater than the other two tissues at 10μM ADP (**Figure 3L**). An improvement in phosphorylation efficiency may be adaptive in DEN-NT tissue, which would likely need to compete with adjacent tumor tissue for nutrients. Notably, DEN-NT also had greater relative expression of all respiratory complexes except for complex IV (CIV) compared to Saline mitochondria, and greater relative expression of CII and complex III than DEN-T (**Supplementary Figure 2J**).

Mitochondrial calcium uptake is an essential mitochondrial function that has been linked to cell fate decisions (e.g., apoptosis and survival). To evaluate whether tumor status affects this ability, we included the fluorometry-based calcium retention assay in our interrogation of DEN-induced HCC. To eliminate the influence of any complex I deficiency, mitochondria were energized with S/Rot in the presence of the HK clamp and 50μM ADP, then calcium was titrated in 30μM increments until the mitochondrial permeability transition pore (MPTP) opened (representative trace in **Figure 3M**; black arrows represent calcium additions). Maximal calcium uptake was similar between Saline and DEN-NT mitochondria, but DEN-T mitochondria were only able to take up roughly 1/3 of the calcium of the nontumor tissues prior to MPTP opening (**Figure 3N**).

### Complex I-Linked Respiratory Deficiency Is Present in DEN-Induced Tumor Mitochondria

**Figure 4:**
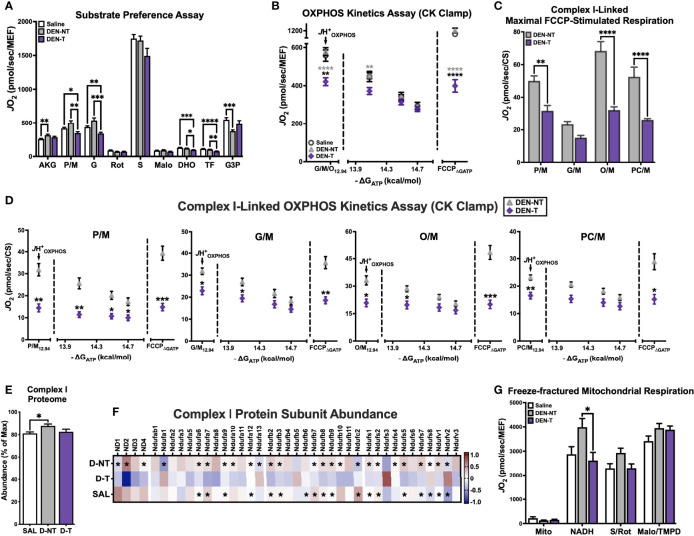
DEN tumor-derived mitochondria exhibit complex I-linked respiratory deficiency. **(A)** Substrate preference assay stimulated with 500μM ADP clamped by HK clamp. Substrate/inhibitor additions were as follows: AKG (10mM), P/M (5/1mM), G (10mM), Rot (0.5μM), S (10mM), Malo (20mM), DHO (10mM), TF (20μM), G3P (5mM). **(B)** OXPHOS kinetics protocol fueled by G/M/O. **(C)** Maximal FCCP-stimulated rates when fueled by 4 different complex I-linked carbon substrates (P/M, G/M, O/M, PC/M); data normalized to CS activity. **(D)** OXPHOS kinetics protocols fueled by P/M, G/M, O/M, PC/M; data normalized to CS activity. **(E)** Relative abundance of complex I subunits expressed as % of highest summed abundance. **(F)** Heatmap of complex I protein subunit expression; asterisks denote significant difference from DEN-T (q<0.1). **(G)** Respiration in freeze-fractured mitochondrial preparations. Data are presented as mean ± SEM and analyzed by two-way ANOVA **(A–D, G)** and one-way ANOVA **(E)**. *p < 0.05, **p < 0.01, ***p < 0.001, ****p < 0.0001.

Until this point, our assessment of DEN-induced tumor mitochondria has consisted of maximal capacity assays under saturating levels of carbon substrates that would feed electrons to multiple sites across the ETS. Although this strategy is effective for identifying broad mitochondrial deficiencies, it may obscure subtle but meaningful differences in the regulation of substrate utilization. We next shifted our focus to evaluating mitochondrial function under varying combinations of respiratory challenges and substrate conditions, beginning with a generalized substrate preference assay (**Figure 4A**).

Mitochondria were energized with saturating ADP (500μM) maintained by the HK clamp, then exposed to a series of carbon substrates and corresponding inhibitors. CI-linked substrates were layered in first, including α-ketoglutarate (AKG), P/M, and G. When fueled by AKG, DEN-T mitochondria were able to match the *J*O_2_ of both nontumor tissues, though respiration was greater in DEN-NT than Saline mitochondria (‘AKG’, **Figure 4A**). The addition of P/M normalized respiration between Saline and DEN-NT, but it stimulated no further increase in DEN-T mitochondria, leading to a significantly lower *J*O_2_ (‘P/M’, **Figure 4A**). Subsequent addition of G did not lead to a meaningful increase in respiration for any of the tissues, and the cumulative CI-supported respiration was greater in Saline and DEN-NT mitochondria than DEN-T (‘G’, **Figure 4A**). NADH-linked respiration was inhibited using Rot, and S was then added to initiate CII-supported flux. Consistent with our findings in cells, respiration fueled by S was not different between tumor and nontumor mitochondria (‘S’, **Figure 4A**). The competitive CII inhibitor malonate (Malo) was then added, followed by dihydroorotate (DHO). DHO is the substrate for dihydroorotate dehydrogenase (DHODH), which feeds electrons directly into the quinone (Q) pool of the ETS from the outer surface of the inner mitochondrial membrane and plays an important role in pyrimidine nucleotide biosynthesis. Although it might be expected that nucleotide synthesis would be important to growing tumors, here DHO-supported respiration was significantly decreased in DEN-T mitochondria compared to both nontumor tissues (‘DHO’, **Figure 4A**). This difference was still present after the addition of DHODH inhibitor teriflunomide (TF), the implications of which are unclear (‘TF’, **Figure 4A**). Finally, respiration was stimulated with a combination of calcium and glycerol-3-phosphate (G3P), which similarly feeds electrons directly into the Q pool through glycerol-3-phosphate dehydrogenase (GPDH). There was no difference between tumor and nontumor G3P-supported respiration, though Saline was able to respire at a higher rate than DEN-NT (‘G3P’, **Figure 4A**).

As there was a consistent decrease in CI-supported respiration seen in both the HCC cell model and DEN-induced tumors, we decided to explore this phenotype further with additional respiratory stimuli. First, we repeated the OXPHOS kinetics assay experiments with G/M/O provided as carbon substrates. This combination was chosen because the contribution of G was hidden within the other CI substrates in the substrate preference assay, and lipid substrates such as O had been excluded. In contrast to the robust *J*H^+^_OXPHOS_ attained by DEN-T under a multi-complex stimulating substrate condition (P/M/G/S/O), the G/M/O-supported *J*H^+^_OXPHOS_ was significantly lower compared to both nontumor tissues (**Figure 4B**). Although by the end of the ΔG_ATP_ titration respiration was equivalent between all tissues, DEN-T mitochondria were again less responsive to FCCP in the presence of ΔG_ATP_ (‘FCCP_ΔGATP_’, **Figure 4B**). Both Saline and DEN-NT tissues were able to double their G/M/O-supported *J*H_OXPHOS_ following FCCP titration, but DEN-T mitochondria were only able to match it (**Figure 4B**).

Although there were striking differences in CI-linked respiration between DEN-T and nontumor mitochondria present in both the substrate preference and G/M/O OXPHOS kinetics assays, it was still unclear whether these differences reflected a global CI deficiency or a deficiency in other enzymatic processes (i.e., dehydrogenase activity, beta-oxidation). To address this limitation, a small subset of DEN-treated mice was used to assess FCCP- and ΔG_ATP_-stimulated respiration under 4 substrate conditions: P/M, G/M, O/M, and palmitoyl-carnitine (PC)/M. This mixture of substrate conditions spanned the most common CI-linked metabolism products of glucose, glutamine, and fatty acid oxidation that may be utilized for mitochondrial respiration. Only DEN-NT and DEN-T tissues were considered for this evaluation as Saline mitochondrial respiration was typically equivalent to DEN-NT. Data from this later subset of mice was normalized to CS activity rather than MEF, as it was found that CS activity from several of these mitochondrial preparations was higher than the averages from earlier in the study (data not shown). When stimulated with FCCP, respiration supported by P/M, O/M, and PC/M substrates were all significantly greater in DEN-NT mitochondria than DEN-T, though there was no difference between tumor and nontumor for the G/M condition (**Figure 4C**). Indeed, both tissues achieved their lowest FCCP-stimulated *J*O_2_ when G/M was used to fuel respiration (**Figure 4C**).

To determine whether ATP free energy had any effect on CI substrate utilization, the same 4 substrate conditions were then used in conjunction with the CK-based OXPHOS kinetics assay as described above (**Figure 4D**). Consistent with limited CI-linked flux in DEN-T mitochondria, *J*H^+^_OXPHOS_ was reduced under all substrate conditions compared to DEN-NT (**Figure 4D**). OXPHOS kinetics appeared most impaired in DEN-T mitochondria when fueled with P/M, as this was the only substrate condition in which respiration was significantly lower across the entire ΔG_ATP_ titration. Additionally, as previously seen with the multi-substrate (P/M/G/S/O) and G/M/O kinetic clamps, FCCP_ΔGATP_ was significantly decreased in DEN-T mitochondria for all conditions.

Together, the data from each of these assays strongly suggested that DEN-T mitochondria had a lower capacity to support respiration using CI-linked carbon substrates, though the nature of this limitation remained unclear. Given that we had previously observed comparable NAD^+^/NADH redox poise between tumor and nontumor tissues (**Figure 3H**), we did not expect the issue to be within the matrix dehydrogenase network. The next possible explanation would be differential expression of the complex itself. We returned to our mitochondrial proteomics data and used the summed abundances of all identified CI subunits to estimate the relative expression of CI in Saline, DEN-NT, and DEN-T mitochondria (**Figure 4E**). Although relative CI expression was found to be greater in DEN-NT than Saline mitochondria, there was no difference between DEN-T and the two nontumor tissues (**Figure 4E**). When we compared the abundance of the individual subunits, however, a majority appeared to have decreased expression in DEN-T mitochondria (**Figure 4F**). In fact, of all protein subunits with significant differences in abundance between tumor and nontumor, only 4 were significantly upregulated in DEN-T: Ndufa13, Ndufs3, Ndufs8, and Ndufv2 (**Figure 4F**). Interestingly, 2 of the 4 identified mitochondrial DNA-encoded CI subunits, ND2 and ND3, which are integral to the catalytic activity of CI, were found to have significantly decreased expression in DEN-T mitochondria compared to DEN-NT (**Figure 4F**).

The functional implications of altering the expression of individual subunits within mitochondrial respiratory complexes have not been well defined to date. We decided to directly test the respiratory capacity of CI in tumor and nontumor mitochondria using a recently published method for respiratory experiments in freeze-fractured mitochondria (29)(**Figure 4G**). Freeze-thawed mitochondria were stimulated using the HK clamp, and NADH, S/Rot, and Malo/TMPD were added sequentially to stimulate CI-, CII-, and CIV-linked respiration, respectively. Intriguingly, NADH-supported respiration was significantly lower in DEN-T mitochondria than DEN-NT, though there was no significant difference between Saline and DEN-T (**Figure 4G**). Consistent with our findings in fresh mitochondria, there were no differences in respiration between any of the groups when fueled by S/Rot or Malo/TMPD.

### Upregulation of Adenylate Kinase 4 in DEN-Induced Tumor Mitochondria Contributes to Altered Complex I-Linked Respiratory Flux

**Figure 5:**
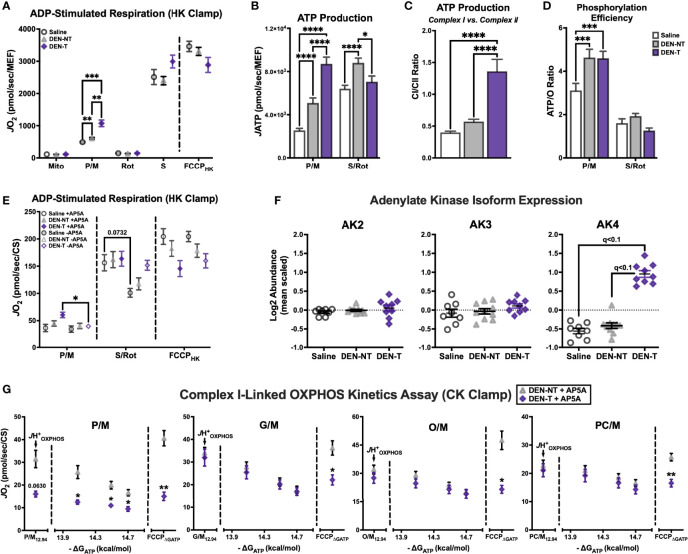
Upregulation of adenylate kinase 4 (AK4) in DEN-induced tumor mitochondria contributes to altered complex I-linked respiratory flux. **(A)** ADP-stimulated protocol fueled by P/M and S/Rot. **(B)** Comparison of *J*ATP fueled by P/M and S/Rot. **(C)** Ratio of CI-supported (P/M) *J*ATP to CII-supported (S/Rot) *J*ATP from **(B)**. **(D)** Comparison of ATP/O ratios fueled by P/M and S/Rot. **(E)** ADP-stimulated protocol fueled by P/M and S/Rot in the presence (+) and absence (-) of adenylate kinase (AK) inhibitor Ap5A; data normalized to CS activity. **(F)** Relative expression of AK isoforms 2-4. **(G)** OXPHOS kinetics protocols in the presence of Ap5A fueled by P/M, G/M, O/M, PC/M; data normalized to CS activity. Data are presented as mean ± SEM and analyzed by two-way ANOVA **(A, B, D, E, G)** and one-way ANOVA **(C)**. *p < 0.05, **p < 0.01, ***p < 0.001, ****p < 0.0001.

Although there was a clear deficit in CI-supported respiration in DEN-T mitochondria, it was still unclear whether this would have a meaningful impact on other important mitochondrial functions such as ATP production. To test this, parallel assessments of respiration and *J*ATP were again conducted under CI-specific (P/M) and CII-specific (S/Rot) substrate conditions and saturating ADP (**Figures 5A, B**). As seen in earlier experiments, S/Rot-supported respiration was similar across all 3 tissues when stimulated by both ADP and FCCP (‘FCCP_HK_’, **Figure 5A**). Unexpectedly, P/M-supported respiration was significantly greater in DEN-T mitochondria compared to both nontumor tissues under these assay conditions (**Figure 5A**). DEN-T mitochondria also generated the greatest *J*ATP of all 3 tissues when fueled by P/M, while DEN-NT mitochondria had the greatest S/Rot-supported *J*ATP (**Figure 5B**). Of note, both Saline and DEN-NT mitochondria were able to attain only about 50% of their S/Rot-supported *J*ATP when fueled by P/M, whereas *J*ATP was nearly equivalent under both conditions for DEN-T mitochondria (**Figure 5C**). Phosphorylation efficiency was greater for both DEN-NT and DEN-T mitochondria compared to Saline under the P/M condition, but there were no differences in efficiency for S/Rot (**Figure 5D**).

The results of this assay demonstrated that ATP production was not compromised in DEN-T mitochondria, though the most surprising outcome was that P/M-supported respiration no longer appeared impaired. This was perplexing, as the respiratory stimulus—500μM ADP—was the same as that used in the substrate preference assay, with seemingly different effects. One important addition that was absent from the substrate preference assay was the adenylate kinase (AK) inhibitor, P1, P5-di(adenosine-5′) pentaphosphate (Ap5A). Ap5A is included in assessments of phosphorylation efficiency as it eliminates ATP production by AK that might confound estimates of oxidative *J*ATP. We repeated the same HK-based respiration assay in the presence and absence of Ap5A and found that P/M-supported respiration was unaffected in Saline and DEN-NT mitochondria, but it was significantly decreased in the absence of Ap5A in DEN-T mitochondria (**Figure 5E**). Conversely, there were no significant effects of Ap5A on FCCP_HK_ in any tissue, though there was a trend for decreased S/Rot-supported respiration in the absence of Ap5A for Saline mitochondria (**Figure 5E**). To understand why DEN-T mitochondria may be particularly sensitive to the presence of Ap5A, we searched our proteomics for expression of AK isoforms. Interestingly, AK4 was found to be overexpressed in DEN-T mitochondria compared to Saline and DEN-NT, while the AK2 and AK3 isoforms were equivalent between the tissues (**Figure 5F**).

Given that Ap5A was able to improve P/M-supported respiration for DEN-T mitochondria stimulated with ADP, we were curious whether Ap5A might similarly improve CI-linked OXPHOS kinetics. Using the same 4 substrate conditions as before—P/M, G/M, O/M, and PC/M—we repeated our CK-clamp based experiments in the presence of Ap5A in DEN-NT and DEN-T mitochondria (**Figure 5G**). Interestingly, Ap5A was able to rescue the *J*H^+^_OXPHOS_ of DEN-T mitochondria to a rate equivalent to DEN-NT mitochondria for all 4 substrates, though the P/M-supported *J*H^+^_OXPHOS_ trended toward respiration being lower in DEN-T (**Figure 5G**). Additionally, for every substrate condition except for P/M, respiration across the ΔG_ATP_ titration was equivalent between DEN-NT and DEN-T. Inclusion of Ap5A did not rescue the FCCP_ΔGATP_, however, and DEN-NT mitochondria were able to attain a significantly higher FCCP-stimulated rate under every substrate condition (**Figure 5G**).

### Human Primary Hepatocellular Carcinoma Tumors Also Display a Reduced Mitochondrial Capacity Compared to Nontumor Liver Tissue

**Figure 6:**
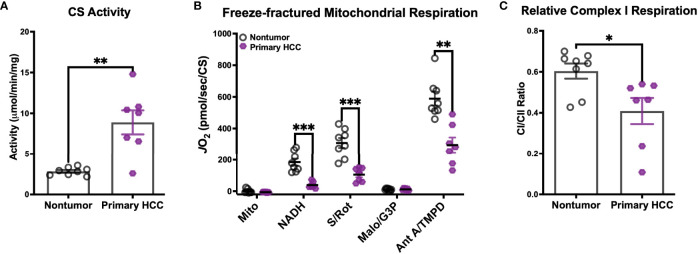
Human primary hepatocellular carcinoma (HCC) tumors display reduced mitochondrial capacity. **(A–C)** Mitochondria were isolated from flash-frozen nontumor liver tissue (Nontumor; n=8) and liver tumor (Primary HCC; n=7) samples from human hepatocellular carcinoma patients for biochemical assessment. **(A)** Citrate synthase (CS) activity of the mitochondrial preparations. **(B)** Respiration in freeze-fractured mitochondria. **(C)** Relative complex I respiration, quantified as the ratio of the NADH-supported JO_2_ to the succinate (S)-supported JO_2_ from **(B)**. Data are presented as mean ± SEM and analyzed by unpaired Student’s t-tests **(A, C)** and two-way ANOVA **(B)**. *p < 0.05, **p < 0.01, ***p < 0.001.

We next sought to determine whether there was evidence of a similar CI-linked respiratory deficiency in the human presentation of hepatocellular carcinoma. Flash-frozen whole tissue samples from a total of 8 HCC patients were obtained through the North Carolina Tissue Consortium. These samples represented a range of cancer stages, defined by the tumor/node/metastasis (TNM) classification system (30), as can be seen in the patient characteristics listed in **Table 1**. Tumor (‘Primary HCC’) and nontumor liver (‘Nontumor’) samples from each patient were used to isolate mitochondria for functional measurements. Mitochondrial purity was determined using the CS activity assay and was found to be higher in the isolates from Primary HCC compared to Nontumor samples (**Figure 6A**).

**Table 1:** Primary hepatocellular carcinoma patient characteristics.

Patient	Cancer Stage	Sex	Age
1	-	M	60-70
2	IVB	M	20-30
3	I	M	50-60
4	I	M	50-60
5	IIIA	M	50-60
6	II	M	50-60
7	II	M	80-90
8	I	F	60-70

Respiration was then evaluated in freeze-fractured mitochondria using the same protocol described above, with the additional inclusion of G3P as a substrate. All respiration rates were normalized to CS activity. Consistent with our findings in DEN-induced tumors, CI-supported respiration (‘NADH’, **Figure 6B**) was depressed in Primary HCC. In fact, respiration rates of Primary HCC mitochondria were lower for all substrate conditions except for G3P, suggesting a global reduction in mitochondrial capacity. To determine whether this diminished capacity disproportionately affected CI, we then calculated the ratio of CI- to CII-supported respiration rates (**Figure 6C**). Although both Nontumor and Primary HCC mitochondria were able to support higher respiration rates with the CII-linked substrate (CI/CII ratios <1), the proportion of that rate that could be stimulated by CI-linked substrate NADH was found to be lower in Primary HCC. This finding is consistent with the reduced CI-supported respiration rates that were observed in DEN-induced HCC tumors in mice.

## Discussion

Through application of our comprehensive mitochondrial diagnostic workflow, we sought to evaluate the functional impact of the metabolic, proteomic, and genomic shifts that have been reported in hepatocellular carcinoma (HCC). Our experiments leveraged a variety of respiratory stimuli and carbon substrate conditions *in vitro*, which allowed us to identify a consistent reduction in CI-supported respiration across two distinct models of HCC—cultured cells and diethylnitrosamine (DEN)-induced tumors—as well as evidence of a reduced respiratory capacity in human primary HCC tumor mitochondria. Further, the pairing of mitochondrial-enriched proteomics and targeted inhibitor use revealed a potential regulatory role of adenylate kinase 4 (AK4) in limiting CI-linked respiratory flux. To our knowledge, this was the first study to combine mitochondrial-targeted proteomics with comprehensive biochemical measures of mitochondrial performance in any model of HCC.

Given previous reports of compromised mitochondrial respiration and reduced expression of respiratory complexes in HCC tumors (19, 20, 22), we expected to find large differences in intrinsic mitochondrial function in the present study. However, both cell culture and DEN models of HCC demonstrated that the total respiratory capacity of HCC was equivalent to or greater than nontumor mitochondria when energized with a mixture of carbon substrates that activated the entire respiratory system (P/M/G/S/O) across multiple respiratory stimuli (uncoupling, clamped ATP free energy, and ADP). This suggests that the manifestation of reduced mitochondrial flux in HCC may not be due to a loss of capacity, but rather an increase in extramitochondrial regulation *in vivo*.

As has been reported by others (19, 20, 22), we found a CI-specific deficit in respiration in both models of HCC. In DEN-induced tumors, this was found to be due to two separate mechanisms: a reduced capacity of CI to directly oxidize NADH, as well as regulation by adenylate kinase activity. The largest of the respiratory complexes, CI is made up of a total of 45 protein subunits encoded in both the nuclear and mitochondrial genomes. The 7 CI genes contained within mitochondrial DNA (mtDNA) are the most hydrophobic and integral to its proper assembly and catalytic activity (31). Interestingly, the reduced functionality of CI in DEN tumors was not linked to an overall loss of abundance of CI-related proteins. However, we did find a specific loss of two mtDNA-encoded subunits, ND2 and ND3, between DEN-induced tumor mitochondria and their adjacent nontumor tissue that may have contributed to a reduced CI capacity. This finding supports those of other groups who have found a high mtDNA mutational burden in HCC (11, 14, 32–34), including the discovery of a mutation in human HCC that would introduce a premature stop codon into the gene for ND1 (11). The loss of these subunits did not appear to affect the ability of the mitochondria to phosphorylate ATP under any combination of respiratory substrates, which may suggest that CI respiratory capacity is in fact separate from phosphorylation capacity. This conclusion has important implications for interpreting mitochondrial respiration data, as oxygen consumption alone may not be a sufficient surrogate for OXPHOS.

DEN-induced tumors were also found to have increased expression of AK4 compared to nontumor tissues. AK4 is a mitochondrial matrix protein that has previously been shown to be upregulated in response to stressors such as hypoxia (35, 36). In support of our findings, AK4 has also been found to be overexpressed in human HCC-derived HepG2 cells (37), as well as other cancer types from a diverse group of tissues (38–42). In cancer, AK4 has been suggested to contribute to progression and metastasis, though the exact mechanism is not clear. Our findings propose that in DEN-induced HCC, AK4 might have a role in regulating respiratory flux through CI, and these effects were particularly pronounced in the presence of an ATP free energy state that mimics physiological demands on mitochondria. It is possible that increased expression of AK4 may lead to a disruption in energy demand signaling, in which consumption of ADP by AK4 to generate ATP would prevent the stimulation of mitochondrial respiration that would normally occur in response to an accumulation of ADP. Our data would support this hypothesis, as inhibition of adenylate kinases by Ap5A was able to restore CI-linked flux for most substrate conditions *in vitro* when the respiratory stimulus was either a bolus of ADP or ATP free energy. Increased regulation of CI may have important implications for mitochondrial metabolism in the context of the whole cell and should be explored further in future investigations. For instance, limiting the oxidation of NADH at CI would presumably slow the oxidation of pyruvate and other NADH-linked energy substrates, leading to the preservation of carbon backbones for biosynthesis, a scenario that has been proposed to be favorable for tumorigenesis (15).

Consistent with our previously published work in leukemia (25), DEN-induced tumor mitochondria displayed a reduced capacity to respond to the uncoupler FCCP in the presence of ATP free energy across all substrate conditions tested. As both leukemic and HCC mitochondria were unable to reattain the respiration rate from earlier in the same assay, it appears that the FCCP effect is due in some part to direct inhibition of the respiratory complexes. In leukemia, this effect was found to be dependent upon the entry of ATP into the mitochondrial matrix, as the addition of the adenine nucleotide translocase (ANT) inhibitor carboxyatractyloside (CAT) restored FCCP-stimulated flux in the presence of ATP free energy (25). Interestingly, we found that CAT had no effect on the FCCP response in HCC tumor mitochondria, suggesting that the effector of the inhibition was not located within the matrix. This finding is intriguing as the same phenotype appears to be caused by two separate mechanisms across the two malignancies, suggesting that it may have an important physiological role in cancer.

We did see limitations to mitochondrial membrane potential (ΔΨ) in DEN-induced tumor mitochondria, consistent with other findings in DEN-treated rodents (19) and our own results in HepG2 cells (28). Depolarization of ΔΨ was evident across a physiological span of ATP free energy despite the availability of all respiratory substrates (P/M/G/S/O). Although surprising, this relative depolarization may explain two other effects that were seen in DEN tumor mitochondria: the reduced K_m_ for FCCP and their reduced ability to retain calcium. In the case of FCCP, respiration is dependent upon the import of respiratory substrates into the mitochondrial matrix, which is dependent upon a polarized inner mitochondrial membrane. As FCCP uncoupling already induces a slight depolarization, the reduced respiration seen in DEN tumor mitochondria stimulated by FCCP was likely caused by a more rapid loss of ΔΨ and subsequent substrate transport.

Similarly, calcium cations require mitochondrial membrane polarization for their accumulation in the matrix, and reduced membrane potential has been linked to increased sensitivity of the mitochondrial transition pore opening (43, 44). The physiological relevance of reduced calcium uptake in HCC is supported by previous reports of elevated cytosolic calcium levels in cell models of HCC with low mitochondrial activity (45). Interestingly, increased intracellular calcium was shown to induce nuclear transcription of NUPR1, a promoter of cancer progression (45). This may imply that low mitochondrial membrane potential and calcium uptake could be a means of retrograde signaling to the nucleus in HCC. This is an interesting avenue to explore further as HCC tumors may be particularly sensitive to treatments designed to manipulate intracellular calcium levels.

Importantly, by leveraging the technique of Acin-Perez et al. (29) to evaluate mitochondrial respiration in previously frozen tissue, we were able to directly assess the mitochondrial capacity of human primary HCC samples. Validating the findings of our cell culture and DEN models, human HCC mitochondria exhibited a reduced capacity for both CI-linked and overall respiratory capacity compared to nontumor liver tissue obtained from the same patients, despite a range of tumor grades across samples. Although this observation is encouraging for the translational relevance of our cell culture and DEN models of HCC, the information that can be gleaned from freeze-fractured mitochondrial respiration is limited. The goal of future studies will be to obtain fresh tumor and nontumor samples of HCC patients to complete the full mitochondrial diagnostic workflow.

Our findings support the utility of mitochondrial phenotyping in identifying novel regulatory mechanisms governing cellular bioenergetics. As all measurements were made in isolated mitochondria, the influence of cytosolic and cytoskeletal regulation on metabolic fluxes was removed to evaluate the intrinsic properties of the mitochondria. Future studies incorporating whole cell or tissue measurements of flux will help to increase understanding of the physiological impact of these differences in HCC.

## Materials and Methods

### Materials and Reagents

Unless indicated, all chemicals and reagents were purchased from Sigma-Aldrich (St. Louis, MO).

### Cell Culture

Mouse hepatocellular carcinoma cells (Hepa 1-6) and mouse immortalized hepatocytes (AML12) were purchased from ATCC (Manassas, VA). Hepa 1-6 cells were cultured in Dulbecco’s Modified Eagle’s Medium (DMEM; Thermo Fisher Scientific, Waltham, MA) containing 4mM L-glutamine, 4.5g/L glucose, 1mM sodium pyruvate, and 1.5g/L sodium bicarbonate, supplemented with 10% fetal bovine serum (FBS; Gibco) and 1% penicillin/streptomycin as recommended by ATCC. AML12 cells were cultured in DMEM: F-12 Medium containing 2.5mM L-glutamine, 15mM HEPES, 0.5mM sodium pyruvate, and 1200mg/L sodium bicarbonate, supplemented with 10% FBS, 1% penicillin/streptomycin, 10μg/ml insulin, 5.5μg/ml transferrin, 5ng/ml selenium, and 40ng/ml dexamethasone.

### Animal Treatment

All animal experiments were conducted in C3H/HeJ mice (The Jackson Laboratory, stock #000,659) according to the guidelines approved by the East Carolina University Institutional Animal Care and Use Committee. Mice were housed under controlled temperature (22.7°C) and light (12h light/12h dark) conditions with free access to food and water. Induction of hepatocellular carcinoma was achieved using a single injection of diethylnitrosamine (DEN), as previously described (46). DEN (25mg/kg) or saline vehicle (0.9% NaCl) was injected intraperitoneally at 14 days postnatal. As mice were not weaned, all littermates received the same treatment. Both DEN (n=23) and saline (n=27) mice were sacrificed after tumor development at ~27 weeks of age. At the time of sacrifice, 12h-fasted mice were anesthetized with isofluorane and exsanguinated prior to tissue removal.

### Human Primary Liver and Tumor Samples

Deidentified, flash-frozen samples of tumor and nontumor liver tissue from human hepatocellular carcinoma patients (n=8) were obtained from the North Carolina Tissue Consortium tissue bank. The use of these samples was considered exempt from approval by the University and Medical Center Institutional Review Board of East Carolina University as none of the 18 HIPPA-protected identifiers were collected for this study.

### Isolation of Mitochondria From Cultured Cells, Mouse Tissues, and Human Tissues

Mitochondrial isolation from cultured cells was performed as previously described (28), with slight modifications. Briefly, cells were trypsinized, centrifuged at 300 x g at room temperature, resuspended in phosphate-buffered saline (PBS), and again centrifuged at 300 x g. This pellet was resuspended in ice-cold Buffer A (50mM MOPS, 100mM KCl, 1mM EGTA, 5mM MgSO_4_, 2g/L bovine serum albumin; pH=7.1) and homogenized *via* a Teflon pestle and borosilicate glass vessel for 40 passes, then centrifuged at 800 x g for 10min at 4°C. The supernatant was saved on ice, and the pellet was resuspended in Buffer A, homogenized, and again centrifuged at 800 x g. Both supernatants were pooled and centrifuged at 10,000 x g for 10min at 4°C. The mitochondrial pellet was then washed in Buffer B (Buffer A with no bovine serum albumin), transferred to a microcentrifuge tube, and centrifuged again at 10,000 x g for 10min at 4°C. Final mitochondrial pellets were resuspended in 100-150μL of Buffer B and protein content was determined *via* the Pierce BCA protein assay.

Livers from DEN- and saline-treated mice were excised, rinsed in ice-cold (4°C) PBS to remove excess blood, and placed on ice in Buffer C (7.23mM K_2_EGTA, 2.77mM CaK_2_EGTA, 20mM imidazole, 20mM taurine, 6.56mM MgCl_2_·6H_2_O, and 50mM K-MES; pH=7.4). Tumors were separated from nontumor adjacent liver tissue under a dissecting microscope. Nontumor tissue was only selected for mitochondrial isolation if there were no visible abnormalities present. Following separation, tissues were transferred to Buffer A, minced, and homogenized *via* Teflon pestle and borosilicate glass for 8 passes, then centrifuged at 800 x g for 10min at 4°C. Supernatants were passed through gauze to remove lipid and then centrifuged at 10,000 x g for 10min at 4°C. This mitochondrial pellet was washed in Buffer B, transferred to a microcentrifuge tube, and again centrifuged at 10,000 x g for 10min. Final mitochondrial pellets were resuspended in 200-300μL of Buffer B and assessed for protein content.

Frozen human primary tissue samples were thawed on wet ice, then suspended in ice-cold Buffer B for homogenization *via* Teflon pestle and borosilicate glass for 8 passes. The resultant homogenate was centrifuged at 1,000 x g for 10min at 4°C. The supernatant was passed through gauze and then centrifuged at 10,000 x g for 10min at 4°C. This pellet was then washed in Buffer B, transferred to a microcentrifuge tube, and spun at 10,000 x g for 10min. Final mitochondrial pellets were resuspended in 100-150μL of Buffer B and assessed for protein content.

### Citrate Synthase Activity

Citrate synthase activity was determined using a plate-based, colorimetric assay as previously described (26). Briefly, citrate synthase activity was quantified using the rate of conversion of 5’, 5’-Dithiobis 2-nitrobenzoic acid (DTNB) to TNB (OD: 412nm) in the presence of 10-20μg mitochondria and 1mM oxaloacetate. Conversion rates were calculated using the Beer-Lambert Law and the molar absorption coefficient of TNB (13.6mM/cm).

### Mitochondrial Respiration Assessment

High-resolution oxygen consumption rate (*J*O_2_) was assessed *via* the Oroboros Oxygraph-2K (Oroboros Instruments, Innsbruck, Austria) as previously described (26), with minor adjustments. Respiration media was Buffer D (105mM K-MES, 30mM KCl, 10mM KH_2_PO_4_, 5mM MgCl_2_, 1mM EGTA, 2.5g/L bovine serum albumin; pH=7.2), with additions as noted. All respiration experiments were conducted in a 1mL reaction volume at 37°C. All respiration assays conducted with cell mitochondria were performed with the addition of 10µM cytochrome C.

A variety of carbon substrates and inhibitors were used to support respiration, with assay-specific substrate conditions defined in figure legends. General substrate concentrations were as follows: pyruvate (P; 5mM), malate (M; 1mM), glutamate (G; 5-10mM), octanoyl-L-carnitine (O; 0.2mM), succinate (S; 5-10mM), palmitoyl-carnitine (PC; 20µM), α-ketoglutarate (AKG; 10mM), dihydroorotate (DHO; 10mM), glycerol-3-phosphate (5mM), calcium (1.2mM), malonate (Malo; 20mM), oligomycin (0.02µM), carbonyl cyanide-4-phenylhydrazone (FCCP; 0.25-4µM), rotenone (Rot; 0.5µM), antimycin A (0.5µM), carboxyatractyloside (CAT, 1µM), 17-AAG (1µM), teriflunomide (TF; 20µM), P1, P5-di(adenosine-5′) pentaphosphate (Ap5A; 0.2mM), creatine kinase (CK; 20U/mL), ATP (5mM), phosphocreatine (PCR; 1mM, 6mM, 15mM, 21mM), hexokinase (HK; 1U/mL), glucose (5mM), ADP (10-500µM).

Electron transport system (ETS) capacity was determined through titration of chemical uncoupler FCCP using 25μg cell mitochondria and 50-100μg tissue mitochondria. Buffer D was supplemented with 5mM creatine monohydrate (Cr), and FCCP was titrated from 0.5-4μM in multi-substrate assays, and from 0.25-4μM for the complex-I specific assays.

Oxidative phosphorylation (OXPHOS) kinetics were determined using the creatine kinase (CK) clamp as previously described (23, 47, 48). The equilibrium constant of the CK reaction (K’_CK_) was used to calculate the free energy of ATP hydrolysis (ΔG_ATP_) in the presence of known quantities of Cr, PCr, and ATP *via* an online resource (https://dmpio.github.io/bioenergetic-calculators/ck_clamp/) described previously (23). Assay buffer was Buffer D supplemented with 5mM Cr, and experiments were conducted using 50μg cell mitochondria and 100-150μg tissue mitochondria. Following the titration of ΔG_ATP_, FCCP was titrated from 0.5-4μM in multi-substrate assays, and from 0.25-4μM for the complex-I specific assays.

The substrate preference assay was conducted using Buffer D supplemented with 5mM Cr and the hexokinase (HK) clamp components (1U/mL HK and 5mM glucose) using 150-200μg tissue mitochondria.

ADP-stimulated respiration assays were performed using Buffer D supplemented with HK (1U/mL), glucose (5mM), glucose-6-phosphate dehydrogenase (2U/mL), and NADP^+^ (4mM), with and without Ap5A. Mitochondrial loading was 50μg cell mitochondria and 100-150μg tissue mitochondria. ADP-linked respiration was inhibited at the end of each assay using oligomycin, then FCCP was titrated from 0.5-3μM. All mitochondrial respiration was inhibited by the addition of complex III-inhibitor antimycin A.

### Mitochondrial NAD^+^/NADH Redox Poise and Membrane Potential (ΔΨ) Assay

NAD^+^/NADH and ΔΨ were determined simultaneously using a QuantaMaster Spectrofluorometer (QM-400, Horiba Scientific, Kyoto, Japan) as previously described (25), with some modifications. Experiments were performed with tissue mitochondria (20µg) in a 200µL reaction volume at 37°C. Assay buffer was Buffer D supplemented with Cr (5mM) and tetramethyl rhodamine methyl ester (TMRM; 0.2 µM). Mitochondria were stimulated using the creatine kinase clamp and ΔG_ATP_ was titrated *via* PCr additions (6, 15, 21mM). Oligomycin (0.02µM) was added to inhibit ATP synthesis, and cyanide (CN, 10mM) was added to induce 100% reduction of the matrix NADH pool, followed by isocitrate (5mM) to induce 100% reduction of the matrix NADPH pool. NAD^+^/NADH was detected at Ex/Em: 350/450 and expressed as a percentage reduction of the CN value as previously described (25). TMRM-derived ΔΨ was quantified by taking the fluorescence ratio of Ex/Em 576/590 to 551/590 and converting this to mV values using a tissue-specific standard curve determined as previously described (23).

### ATP Synthesis Assay

ATP production rate (*J*ATP) was determined fluorometrically using a QuantaMaster Spectrofluorometer (QM-400, Horiba Scientific, Kyoto, Japan) as previously described (26), with minor alterations. Briefly, ATP synthesis assays were performed using Buffer D supplemented with HK (1U/mL), glucose (5mM), glucose-6-phosphate dehydrogenase (2U/mL), NADP^+^ (4mM), and Ap5A. Ap5A, an inhibitor of adenylate kinase, was present to prevent non-OXPHOS-linked generation of ATP (49). Mitochondrial loading was 5-15μg in a reaction volume of 200μL. The rate of NADPH generation was detected using autofluorescence (Ex/Em: 350/450) and was equated to the rate of ATP production as previously described (49). Parallel respiration assays were performed in the Oroboros Oxygraph-2K (Oroboros Instruments, Innsbruck, Austria) to generate ATP/O ratios as previously described (26). Experiments with cell mitochondria were performed with the addition of 10µM cytochrome C.

### Freeze-Fractured Mitochondrial Respiration

Experiments using freeze-fractured mitochondria from mouse and human primary tissues were performed using a protocol adapted from Acin-Perez et al. (29). Mitochondrial pellets were frozen on dry ice, then thawed on wet ice and resuspended in Buffer B. Protein concentration was determined using a Pierce BCA assay. Respiration assay buffer was Buffer D supplemented with 5mM creatine, 2mM NAD^+^, 0.1mM coenzyme A, 0.3mM thiamine pyrophosphate, 10µM cytochrome C, 1U/L HK, and 5mM glucose. Mitochondria (40-50µg) were added to the chamber, followed by ADP (500µM). Complex I-linked respiration was stimulated with NADH (2mM), then inhibited with Rot (0.5µM). Complex II-linked respiration was then stimulated with S (10mM) and inhibited by Malo (20mM), followed by antimycin A (0.5µM) to inhibit complex III. Complex IV-linked respiration was then stimulated with N,N,N’,N’-tetramethyl-*p*-phenylenediamine (TMPD; 0.5mM dissolved in 2mM ascorbate) and ascorbate (2mM). Respiration in the presence of inhibitors was considered to be non-mitochondrial and subtracted to obtain final respiration rates (ex. Final NADH-stimulated *J*O_2_ = NADH *J*O_2_ – Rot *J*O_2_).

### Calcium Retention Assay

Calcium retention was evaluated fluorometrically using a QuantaMaster Spectrofluorometer (QM-400, Horiba Scientific, Kyoto, Japan) in a 200µL reaction volume at 37°C as previously described (34), with some modification. Assay buffer was Buffer E (0.25M sucrose, 10mM Trizma-HCl, 20mM Trizma-base, 10mM KH_2_PO_4_, 0.5mg/mL bovine serum albumin, 5mM Cr, 40µM EGTA, 1U/mL HK, 5mM glucose; pH=7.1) supplemented with 1µM calcium green 5N to fluorescently measure extracellular calcium (Ex/Em: 506/532). Mitochondria (50µg) were energized with S/Rot and 50µM ADP, then calcium was titrated in 30µM additions until mitochondrial permeability transition pore (MPTP) opening (rapid increase in fluorescence trace). Additional 100µM and 30µM additions were made following MPTP opening to confirm no additional calcium uptake. The assay was then ended with the addition of 10mM EGTA. Calcium retention capacity was considered the amount of calcium added prior to MPTP opening.

### nLC-MS/MS for Label-Free Proteomics

Mitochondria were lysed, digested, and lyophilized for proteomics as previously described (26). Briefly, mitochondrial pellets were lysed in Buffer F (8M urea in 40mM Tris, 30mM NaCl, 1mM CaCl_2_, 1 cOmplete ULTRA mini EDTA-free protease inhibitor tablet; pH=8.0) through two freeze-thaw cycles and sonication for 5s at an amplitude of 30 using a probe sonicator (Q Sonica #CL-188). Equal amounts of protein (200µg) were then reduced with 5mM dithiothreitol (30min incubation at 32°C), alkylated with 15mM iodoacetamide (30min incubation in the dark at room temperature), and then unreacted iodoacetamide was quenched with an additional 10mM dithiothreitol. Digestion was performed in two steps: first using Lys C (ThermoFisher, Cat#90307; 1:100 w:w enzyme:protein; 4h incubation at 32°C), followed by dilution of the samples to 1.5M urea using 40mM Tris (pH=8.0), 30mM NaCl, 1mM CaCl_2_ for overnight digestion *via* trypsin at 32°C (Promega, Cat# V5113; 1:50 w:w enzyme:protein). Following digestion, samples were acidified to 0.5% TFA, centrifuged at 10,000 x g for 10min to pellet undigested material, and the supernatant was collected for desalting of soluble peptides using 50mg tC18 SEP-PAK solid phase extraction columns (Waters; Cat# WAT054955) as previously described (50). The resulting eluate was frozen on dry ice and lyophilized.

Final peptides were resuspended in 0.1% formic acid for peptide quantification (ThermoFisher Cat# 23275) and dilution to a final concentration of 0.25µg/µL. nanoLC-MS/MS analysis was performed using an UltiMate 3000 RSLCnano system (ThermoFisher) coupled to a Q Exactive Plus Hybrid Quadrupole-Orbitrap mass spectrometer (ThermoFisher) *via* a nanoelectrospray ionization source as previously described (26). MS1 was performed at a resolution of 70,000, with an AGC target of 3x10^6^ ions and a maximum injection time (IT) of 100ms. Data-dependent acquisition (DDA) was used to collect MS2 spectra of the top 15 most abundant precursor ions with a charge >1 per MS1 scan, with dynamic exclusion enabled for 20s. The isolation window for precursor ions was 1.5m/z and normalized collision energy was 27. MS2 scans were performed at 17,500 resolution, maximum IT of 50ms, and AGC target of 1x10^5^ ions.

### Data Analysis for Label-Free Proteomics

Proteome Discoverer 2.2 (PDv2.2) was used for raw data analysis and principal component analysis. Default search parameters included oxidation as a variable modification and carbamidomethyl (57.021 Da on C) as a fixed modification. Data were searched against both the Uniprot Mus musculus reference proteome (Proteome ID: UP000000589) and mouse Mito Carta 3.0 database (27). As previously described (26), PSMs were filtered to a 1% FDR and grouping to unique peptides was also maintained at a 1% FDR at the peptide level. Strict parsimony was used to group peptides to proteins, and proteins were again filtered to 1% FDR. MS1 precursor intensity was used for peptide quantification, and low abundance resampling was used for imputation. As previously described (26), high confidence master proteins were used to determine mitochondrial enrichment factor (MEF) by quantifying the ratio of mitochondrial protein abundance (identified using the MitoCarta 3.0 database) to total protein abundance.

### Statistical Analysis

Statistical analysis of label-free proteomics was performed as described previously (26). Briefly, all raw mass spectrometry data were normalized to total protein abundance, converted to the Log_2_ space, then compared between groups using two-tailed Student’s t-tests with a Benjamini Hochberg FDR correction for multiple comparisons. Statistical significance for the adjusted p-value (denoted as q throughout this manuscript) was set to q<0.1. All functional assays were analyzed using Prism 9, and the results are all presented as mean ± SEM (error bars). Data were normalized to mitochondrial enrichment factor (MEF), as previously described (26), or citrate synthase (CS) activity, as indicated in individual figures. Statistical significance was set at p<0.05, and details of statistical analysis are included within figure legends.

## Data Availability Statement

The datasets presented in this study can be found in online repositories. The name of the repository and accession number can be found below: jPOST Proteome Xchange; PXD033362/JPST001567.

## Ethics Statement

The animal study was reviewed and approved by East Carolina University Institutional Animal Care and Use Committee.

## Author Contributions

KM was responsible for study conceptualization, data curation, investigation, methodology, formal analysis, data visualization, writing the original draft of the manuscript as well as revisions and edits. MN, HC, JH, MM, were responsible for study conceptualization, data curation, investigation, methodology, and reviewing and editing of the manuscript. AW and TZ were responsible for data curation, methodology, and reviewing and editing the manuscript. NV was responsible for resources and reviewing and editing the manuscript. KF-W. was responsible for study conceptualization, resources, data curation, methodology, supervision, funding acquisition, and reviewing and editing the manuscript. All authors contributed to the article and approved the submitted version.

## Funding

This project was funded using intramural funding from East Carolina University.

## Conflict of Interest

The authors declare that the research was conducted in the absence of any commercial or financial relationships that could be construed as a potential conflict of interest.

## Publisher’s Note

All claims expressed in this article are solely those of the authors and do not necessarily represent those of their affiliated organizations, or those of the publisher, the editors and the reviewers. Any product that may be evaluated in this article, or claim that may be made by its manufacturer, is not guaranteed or endorsed by the publisher.

## References

[1] Siegel RL Miller KD Fuchs HE Jemal A. Cancer Statistics, 2021. CA: A Cancer J Clin (2021) 71(1):7–33. doi: 10.3322/caac.21654 33433946

[2] Villanueva A. Hepatocellular Carcinoma. New Engl J Med (2019) 380(15):1450–62. doi: 10.1056/NEJMra1713263 30970190

[3] Forner A Llovet JM Bruix J. Hepatocellular Carcinoma. Lancet (2012) 379(9822):1245–55. doi: 10.1016/S0140-6736(11)61347-0 PMC13537732 22353262

[4] Lee HY Nga HT Tian J Yi HS. Mitochondrial Metabolic Signatures in Hepatocellular Carcinoma. Cells (2021) 10(8):1–15. doi: 10.3390/cells10081901 PMC8391498 34440674

[5] García-Chávez JN Vásquez-Garzón VR López MG Villa-Treviño S Montiel R. Integration of Chronological Omics Data Reveals Mitochondrial Regulatory Mechanisms During the Development of Hepatocellular Carcinoma. PloS One (2021) 16(8):e0256016. doi: 10.1371/journal.pone.0256016 34383828 PMC8360386

[6] Cassim S Raymond VA Lacoste B Lapierre P Bilodeau M. Metabolite Profiling Identifies a Signature of Tumorigenicity in Hepatocellular Carcinoma. Oncotarget (2018) 9(42):26868–83. doi: 10.18632/oncotarget.25525 PMC6003570 29928490

[7] Lee NCW Carella MA Papa S Bubici C. High Expression of Glycolytic Genes in Cirrhosis Correlates With the Risk of Developing Liver Cancer. Front Cell Dev Biol (2018) 6:138. doi: 10.3389/fcell.2018.00138 30430110 PMC6220322

[8] Lee Y-K Lim JJ Jeoun U-W Min S Lee E-B Kwon SM. Lactate-Mediated Mitoribosomal Defects Impair Mitochondrial Oxidative Phosphorylation and Promote Hepatoma Cell Invasiveness. J Biol Chem (2017) 292(49):20208–17. doi: 10.1074/jbc.M117.809012 PMC5724007 28978646

[9] Yamada S Nomoto S Fujii T Kaneko T Takeda S Inoue S. Correlation Between Copy Number of Mitochondrial DNA and Clinico-Pathologic Parameters of Hepatocellular Carcinoma. Eur J Surg Oncol (2006) 32(3):303–7. doi: 10.1016/j.ejso.2006.01.002 16478656

[10] Yin PH Lee HC Chau GY Wu YT Li SH Lui WY. Alteration of the Copy Number and Deletion of Mitochondrial DNA in Human Hepatocellular Carcinoma. Br J Cancer (2004) 90(12):2390–6. doi: 10.1038/sj.bjc.6601838 PMC2409531 15150555

[11] Yin PH Wu CC Lin JC Chi CW Wei YH Lee HC. Somatic Mutations of Mitochondrial Genome in Hepatocellular Carcinoma. Mitochondrion [Internet] (2010) 10(2):174–82. doi: 10.1016/j.mito.2009.12.147 20006738

[12] Lee HC Li SH Lin JC Wu CC Yeh DC Wei YH. Somatic Mutations in the D-Loop and Decrease in the Copy Number of Mitochondrial DNA in Human Hepatocellular Carcinoma. Mutat Res (2004) 547:71–8. doi: 10.1016/j.mrfmmm.2003.12.011 15013701

[13] Corral M Kitzis A Baffet G Paris B Tichonicky L Kruh J. RNAs Containing Mitochondrial ND6 and COI Sequences Present an Abnormal Structure in Chemically Induced Rat Hepatomas. Nucleic Acids Res (1989) 17(13):5191–206. doi: 10.1093/nar/17.13.5191 PMC318105 2548155

[14] Corral M Paris B Baffet G Tichonicky L Guguen-Guillouzo C Kruh J. Increased Level of the Mitochondrial ND5 Transcript in Chemically Induced Rat Hepatomas. Exp Cell Res (1989) 184(1):158–66. doi: 10.1016/0014-4827(89)90374-1 2507335

[15] vander Heiden MG Cantley LC Thompson CB. Understanding the Warburg Effect: The Metabolic Requirements of Cell Proliferation. Sci (1979). (2009) 324(5930):1029–33. doi: 10.1126/science.1160809 PMC2849637 19460998

[16] Chen Z Li S Shen M Lu X Bao C Chen D. The Mutational and Transcriptional Landscapes of Hepatocarcinogenesis in a Rat Model. iScience (2020) 23(11):101690. doi: 10.1016/j.isci.2020.101690 33163943 PMC7600387

[17] Wurmbach E Chen YB Khitrov G Zhang W Roayaie S Schwartz M. Genome-Wide Molecular Profiles of HCV-Induced Dysplasia and Hepatocellular Carcinoma. Hepatol (2007) 45(4):938–47. doi: 10.1002/hep.21622 17393520

[18] Aleksic K Lackner C Geigl JB Schwarz M Auer M Ulz P. Evolution of Genomic Instability in Diethylnitrosamine-Induced Hepatocarcinogenesis in Mice. Hepatol (2011) 53(3):895–904. doi: 10.1002/hep.24133 21374661

[19] Chavez E Lozano-Rosas MG Dominguez-Lopez M Velasco-Loyden G Rodriguez-Aguilera JR Jose-Nunez C. Functional, Metabolic, and Dynamic Mitochondrial Changes in the Rat Cirrhosis-Hepatocellular Carcinoma Model and the Protective Effect of IFC-305. J Pharmacol Exp Ther (2017) 361:292–302. doi: 10.1124/jpet.116.239301 28209723

[20] Boitier E Merad-Boudia M Guguen-Guillouzo C Defer N Ceballos-Picot I Leroux JP. Impairment of the Mitochondrial Respiratory Chain Activity in Diethylnitrosamine-Induced Rat Hepatomas: Possible Involvement of Oxygen Free Radicals. Cancer Res (1995) 55(14):3028–35. 7606723

[21] Suresh V Anbazhagan C Thangam R Senthilkumar D Senthilkumar N Kannan S. Stabilization of Mitochondrial and Microsomal Function of Fucoidan From Sargassum Plagiophyllum in Diethylnitrosamine Induced Hepatocarcinogenesis. Carbohydr Polymers [Internet] (2013) 92(2):1377–85. doi: 10.1016/j.carbpol.2012.10.038 23399167

[22] Santos NP Pereira IVOC Pires MJ Lopes C Andrade R Oliveira MM. Histology, Bioenergetics and Oxidative Stress in Mouse Liver Exposed to N-Diethylnitrosamine. In Vivo (2012) 26(6):921 LP – 929. 23160673

[23] Fisher-Wellman K Davidson M Narowski T Lin C-T Koves TR Muoio DM. Mitochondrial Diagnostics: A Multiplexed Assay Platform for Comprehensive Assessment of Mitochondrial Energy Fluxes. Cell Rep (2018) 24:3593–606. doi: 10.1016/j.celrep.2018.08.091 PMC6237617 30257218

[24] Fisher-Wellman KH Draper JA Davidson MT Williams AS Narowski TM Slentz DH. Respiratory Phenomics Across Multiple Models of Protein Hyperacylation in Cardiac Mitochondria Reveals a Marginal Impact on Bioenergetics. Cell Rep (2019) 26(6):1557–1572.e8. doi: 10.1016/j.celrep.2019.01.057 30726738 PMC6478502

[25] Nelson MAM McLaughlin KL Hagen JT Coalson HS Schmidt C Kassai M. Intrinsic Oxphos Limitations Underlie Cellular Bioenergetics in Leukemia. Elife (2021) 10:1–31. doi: 10.7554/eLife.63104 PMC8221809 34132194

[26] McLaughlin KL Hagen JT Coalson HS Nelson MAM Kew KA Wooten AR. Novel Approach to Quantify Mitochondrial Content and Intrinsic Bioenergetic Efficiency Across Organs. Sci Rep (2020) 10(1):1–15. doi: 10.1038/s41598-020-74718-1 33077793 PMC7572412

[27] Rath S Sharma R Gupta R Ast T Chan C Durham TJ. MitoCarta3.0: An Updated Mitochondrial Proteome Now With Sub-Organelle Localization and Pathway Annotations. Nucleic Acids Res (2021) 49(D1):D1541–7. doi: 10.1093/nar/gkaa1011 PMC7778944 33174596

[28] Schmidt CA McLaughlin KL Boykov IN Mojalagbe R Ranganathan A Buddo KA. Aglycemic Growth Enhances Carbohydrate Metabolism and Induces Sensitivity to Menadione in Cultured Tumor-Derived Cells. Cancer Metab (2021) 9(1):1–21. doi: 10.1186/s40170-021-00241-0 33468237 PMC7816515

[29] Acin-Perez R Benador IY Petcherski A Veliova M Benavides GA Lagarrigue S. A Novel Approach to Measure Mitochondrial Respiration in Frozen Biological Samples. EMBO J (2020) 39(13):e104073. doi: 10.15252/embj.2019104073 32432379 PMC7327496

[30] Amin MB Greene FL Edge SB Compton CC Gershenwald JE Brookland RK. The Eighth Edition AJCC Cancer Staging Manual: Continuing to Build a Bridge From A Population-Based to a More “Personalized” Approach to Cancer Staging. CA: Cancer J Clin (2017) 67:93–9. doi: 10.3322/caac.21388 28094848

[31] Falkenberg M Larsson N-G Gustafsson CM. DNA Replication and Transcription in Mammalian Mitochondria. Annu Rev Biochem (2007) 76:679–99. doi: 10.1146/annurev.biochem.76.060305.152028 17408359

[32] Hsu CC Lee HC Wei YH. Mitochondrial DNA Alterations and Mitochondrial Dysfunction in the Progression of Hepatocellular Carcinoma. World J Gastroenterol (2013) 19(47):8880–6. doi: 10.3748/wjg.v19.i47.8880 PMC3870539 24379611

[33] Lee HC Chang CM Chi CW. Somatic Mutations of Mitochondrial DNA in Aging and Cancer Progression. Ageing Res Rev (2010) 9(SUPPL.):S47–58. doi: 10.1016/j.arr.2010.08.009 20816876

[34] Sloan RC Moukdar F Frasier CR Patel HD Bostian PA Lust RM. Mitochondrial Permeability Transition in the Diabetic Heart: Contributions of Thiol Redox State and Mitochondrial Calcium to Augmented Reperfusion Injury. J Mol Cell Cardiol [Internet] (2012) 52(5):1009–18. doi: 10.1016/j.yjmcc.2012.02.009 22406429

[35] Chen L Fink T Ebbesen P Zachar V. Temporal Transcriptome of Mouse ATDC5 Chondroprogenitors Differentiating Under Hypoxic Conditions. Exp Cell Res (2006) 312(10):1727–44. doi: 10.1016/j.yexcr.2006.02.013 16580664

[36] Wujak M Wilke T Weiss A Brosien M Wu C-Y Schermuly RT. Role of Adenylate Kinase 4 as a New Metabolic Regulator of Pulmonary Artery Smooth Muscle Cells Under Hypoxia. Eur Respir J (2019) 54(suppl 63):PA5044. doi: 10.1183/13993003.congress-2019.PA5044

[37] Kong F Binas B Moon JH Kang SS Kim HJ. Differential Expression of Adenylate Kinase 4 in the Context of Disparate Stress Response Strategies of HEK293 and HepG2 Cells. Arch Biochem Biophys (2013) 533(1):11–7. doi: 10.1016/j.abb.2013.02.014 23474458

[38] Zhang J Yin YT Wu CH Qiu RL Jiang WJ Deng XG. AK4 Promotes the Progression of HER2-Positive Breast Cancer by Facilitating Cell Proliferation and Invasion. Dis Markers (2019) 2019. doi: 10.1155/2019/8186091 PMC6886328 31827645

[39] Jan Y-H Lai T-C Yang C-J Lin Y-F Huang M-S Hsiao M. Adenylate Kinase 4 Modulates Oxidative Stress and Stabilizes HIF-1α to Drive Lung Adenocarcinoma Metastasis. J Hematol Oncol (2019) 12(1):12. doi: 10.1186/s13045-019-0698-5 30696468 PMC6352453

[40] Fujisawa K Terai S Takami T Yamamoto N Yamasaki T Matsumoto T. Modulation of Anti-Cancer Drug Sensitivity Through the Regulation of Mitochondrial Activity by Adenylate Kinase 4. J Exp Clin Cancer Res (2016) 35(1):1–15. doi: 10.1186/s13046-016-0322-2 26980435 PMC4793738

[41] Xin F Yao D-W Fan L Liu J-H Liu X-D. Adenylate Kinase 4 Promotes Bladder Cancer Cell Proliferation and Invasion. Clin Exp Med (2019) 19(4):525–34. doi: 10.1007/s10238-019-00576-5 31463832

[42] Jan Y-H Tsai H-Y Yang C-J Huang M-S Yang Y-F Lai T-C. Adenylate Kinase-4 Is a Marker of Poor Clinical Outcomes That Promotes Metastasis of Lung Cancer by Downregulating the Transcription Factor Atf3. Cancer Res (2012) 72(19):5119 LP – 5129. doi: 10.1158/0008-5472.CAN-12-1842 23002211

[43] Nicholls DG. Mitochondria and Calcium Signaling. Cell Calcium (2005) 38(3):311–7. doi: 10.1016/j.ceca.2005.06.011 16087232

[44] Camara Y Mampel T Armengol J Villarroya F Dejean L. UCP3 Expression in Liver Modulates Gene Expression and Oxidative Metabolism in Response to Fatty Acids, and Sensitizes Mitochondria to Permeability Transition. Cell Physiol Biochem (2009) 24(3–4):243–52. doi: 10.1159/000233249 19710539

[45] Lee Y-K Jee BA Kwon SM Yoon Y-S Xu WG Wang H-J. Identification of a Mitochondrial Defect Gene Signature Reveals NUPR1 as a Key Regulator of Liver Cancer Progression. Hepatology (2015) 62(4):1174–89. doi: 10.1002/hep.27976 PMC6312643 26173068

[46] Tolba R Kraus T Liedtke C Schwarz M Weiskirchen R. Diethylnitrosamine (DEN)-Induced Carcinogenic Liver Injury in Mice. Lab Animals (2015) 49(S1):59–69. doi: 10.1177/0023677215570086 25835739

[47] Glancy B Barstow T Willis WT. Linear Relation Between Time Constant of Oxygen Uptake Kinetics, Total Creatine, and Mitochondrial Content *In Vitro* . AJP: Cell Physiol (2008) 294(1):C79–87. doi: 10.1152/ajpcell.00138.2007 17942641

[48] Glancy B Willis WT Chess DJ Balaban RS. Effect of Calcium on the Oxidative Phosphorylation Cascade in Skeletal Muscle Mitochondria. Biochemistry (2013) 52(16):2793–809. doi: 10.1021/bi3015983 PMC4157357 23547908

[49] Lark DS Torres MJ Lin C-T Ryan TE Anderson EJ Neufer PD. Direct Real-Time Quantification of Mitochondrial Oxidative Phosphorylation Efficiency in Permeabilized Skeletal Muscle Myofibers. Am J Physiol - Cell Physiol (2016) 311(2):C239–45. doi: 10.1152/ajpcell.00124.2016 PMC5129772 27335172

[50] McLaughlin KL Kew KA McClung JM Fisher-Wellman KH. Subcellular Proteomics Combined With Bioenergetic Phenotyping Reveals Protein Biomarkers of Respiratory Insufficiency in the Setting of Proofreading-Deficient Mitochondrial Polymerase. Sci Rep (2020) 10(1):1–10. doi: 10.1038/s41598-020-60536-y 32107436 PMC7046634

