# Supplementary material for: Bioenergetic Phenotyping of DEN-Induced Hepatocellular Carcinoma Reveals a Link Between Adenylate Kinase Isoform Expression and Reduced Complex I-Supported Respiration
Source: Front Oncol. 2022 Jun 8;12:919880. doi: 10.3389/fonc.2022.919880 (PMC9213884; doi:10.3389/fonc.2022.919880)
Supplement: Supplementary file 1 [file DataSheet_1.pdf]

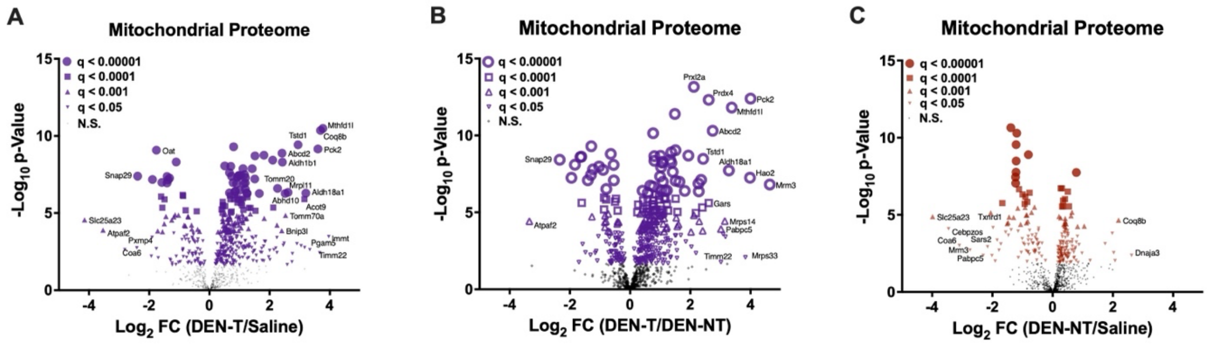

Supplementary Figure 1. Pairwise mitochondrial proteome volcano plot comparisons between mouse liver and tumor tissues. (**A-C**) Volcano plots of differentially expressed mitochondrial proteins identified using the mouse MitoCarta 3.0 database. Plots depict differences between tumor (DEN-T) and saline-treated liver (Saline; **A**), tumor and DEN-treated nontumor liver (DEN-NT; **B**), and DEN-NT and Saline tissues (**C)**. Significance is denoted by the size and color of each symbol, as indicated in the legend. Gray symbols indicate adjusted p value>0.1.

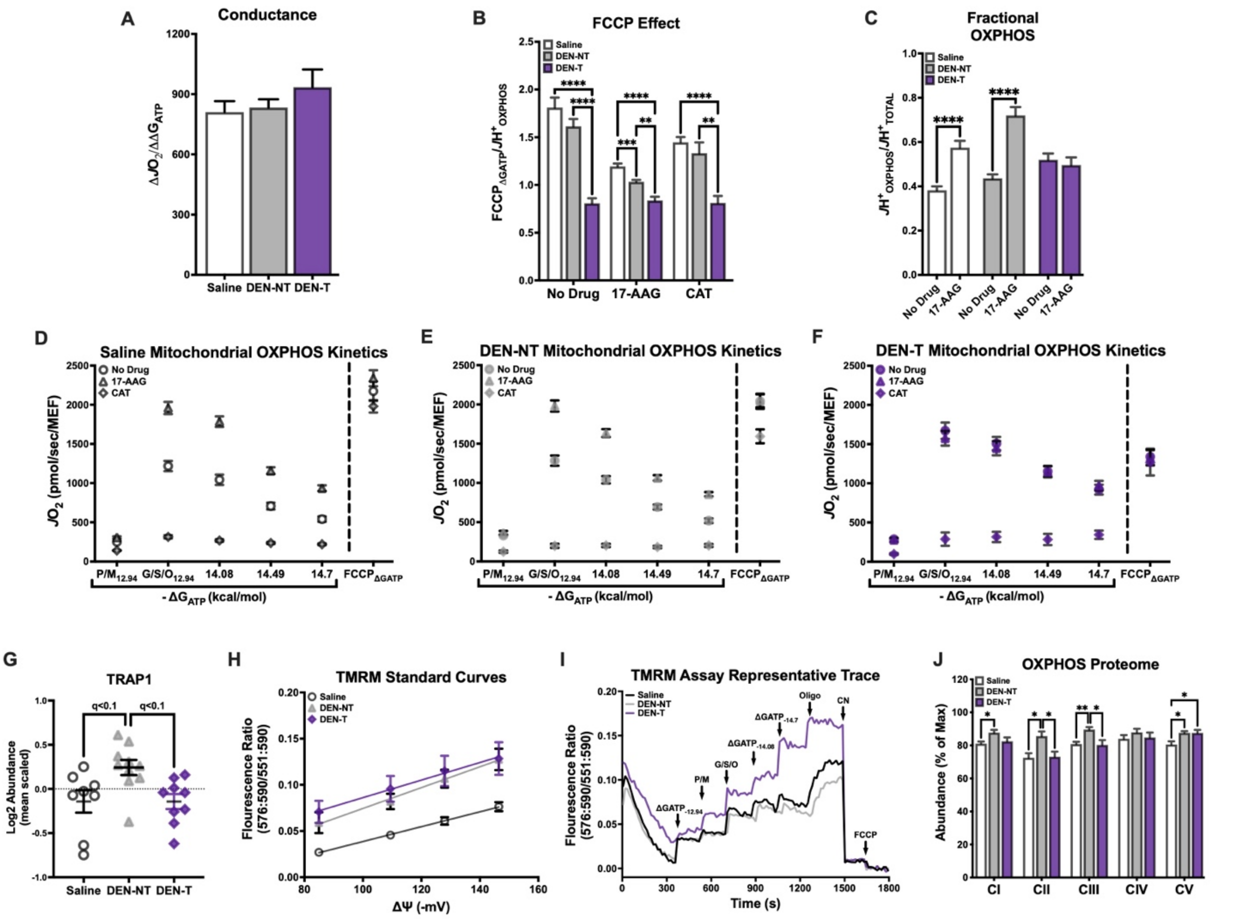

Supplementary Figure 2. Effects of inhibitors 17AAG and CAT on OXPHOS kinetics. (**A**) Comparison of respiratory conductance (dotted lines in **Figure 3E**). (**B**) Comparison of FCCP effect in the presence of no drug, HSP90 inhibitor 17AAG, and adenine dinucleotide transporter inhibitor CAT. (**C**) Comparison of Fractional OXPHOS in the presence and absence of 17AAG. (**D-F**) OXPHOS kinetics protocols in the presence of no drug, 17AAG, or CAT for Saline (**D**), DEN-NT (**E**), and DEN-T (**F**). (**G**) Comparison of relative TRAP1 abundance. (**H**) Plotted TMRM standard curves for Saline, DEN-NT, and DEN-T. (**I**) Representative fluorometric trace of TMRM membrane potential assay. (**J**) Relative abundance of OXPHOS complex protein subunits, presented as % maximal summed abundance per complex. Data are presented as mean ± SEM and analyzed by two-way ANOVA (**B-F, J**) and one-way ANOVA (**A**). *p<0.05, **p<0.01, ***p<0.001, ****p<0.0001.
